# Forms of benefit sharing in global health research undertaken in resource poor settings: a qualitative study of stakeholders' views in Kenya

**DOI:** 10.1186/1747-5341-7-7

**Published:** 2012-01-17

**Authors:** Geoffrey M Lairumbi, Michael Parker, Raymond Fitzpatrick, Michael C English

**Affiliations:** 1 Child and Newborn Health Group, Kenya Medical Research Institute, Centre for Geographic Medicine Research-Coast, Nairobi Unit, PO Box 43640, 00100, Nairobi, Kenya; 2 Department of Public Health, Oxford University, Old Road Campus, Headington, Oxford, OX3 7LF, UK; 3 Ethox Centre, Department of Public Health, Oxford University, Road Campus, Headington, Oxford, OX3 7LF, UK

## Abstract

**Background:**

Increase in global health research undertaken in resource poor settings in the last decade though a positive development has raised ethical concerns relating to potential for exploitation. Some of the suggested strategies to address these concerns include calls for providing universal standards of care, reasonable availability of proven interventions and more recently, promoting the overall social value of research especially in clinical research. Promoting the social value of research has been closely associated with providing fair benefits to various stakeholders involved in research. The debate over what constitutes fair benefits; whether those that addresses micro level issues of justice or those focusing on the key determinants of health at the macro level has continued. This debate has however not benefited from empirical work on what stakeholders consider fair benefits. This study explores practical experiences of stakeholders involved in global health research in Kenya, over what benefits are fair within a developing world context.

**Methods and results:**

We conducted in-depth interviews with key informants drawn from within the broader health research system in Kenya including researchers from the mainstream health research institutions, networks and universities, teaching hospitals, policy makers, institutional review boards, civil society organisations and community representative groups.

The range of benefits articulated by stakeholders addresses both micro and macro level concerns for justice by for instance, seeking to engage with interests of those facilitating research, and the broader systemic issues that make resource poor settings vulnerable to exploitation. We interpret these views to suggest a need for global health research to engage with current crises that face people in these settings as well as the broader systemic issues that produce them.

**Conclusion:**

Global health research should provide benefits that address both the micro and macro level issues of justice in order to forestall exploitation. Embracing the two is however challenging in terms of how the various competing interests/needs should be balanced ethically, especially in the absence of structures to guide the process. This challenge should point to the need for greater dialogue to facilitate value clarification among stakeholders.

## Introduction

The last decade has witnessed increased health related research in resource poor settings mainly in Africa, Asia and South America [1]. While this increase is a positive development in addressing neglected diseases [1-4], it also ushered in new concerns over potential for exploitation through unfair distribution of risks and benefits among the parties involved [5-10]. Several strategies to address these concerns have been promoted in the ethics literature including calls for universal standards of care [11-13], reasonable availability of proven interventions [14-18] and more recently, promotion of the overall social value of research [19]. Closely related to the idea of promoting the social value of research is the determination of fair benefits through the consideration of what, if anything, is owed to those participating in research and their communities, a process that has come to be referred to as benefit sharing [20-24].

Although there now seems an international consensus on the need to share benefits arising from global health research [23,25], there has been continued debate over what constitutes a fair benefit, whether those that address the micro level issues of justice (that is those relating to individual circumstances of those participating in research [26-28] or those focusing on the macro level (that is, the broader issues that might predispose participants to exploitation) [29-32]. The former are often criticised for ignoring the background conditions of justice [30,33] and failing to engage fully with communities. Instead, an alternative view that proposes better engagement with the broader social determinants of health (historical grievances, current political, social and economic forces) that perpetuate poverty and ill health has been proposed [34,35]. Various counter claims include the charge that research does not have a primary aim of restoring inequalities or the provision of health care, and should therefore not be subject to the principles of health care provision [36]. In addition the approach is seen by some as conflating benefits in research participation with those of clinical care [37,38]. Miller for instance [38] has noted that there is a significant ethical difference between the two, and conflating the two can lead to errors in ethical judgment.

Despite the intensity of this debate within ethics of global health research undertaken in resource poor settings, previous empiric work on benefits has almost exclusively focused on micro level issues, and evaluation of alternative benefits [39-42] and there has been less attention to empirically examine the practicalities of these positions on both micro and macro level benefits in real life settings. We aimed to explore the views of stakeholders involved in health related research regarding these forms of benefit sharing in a developing world context.

## Context and methods

This work was part of a larger project examining the concept and practice of benefit sharing in health related research in resource poor settings. Kenya like many other resource poor settings lacks a well functioning research governance framework to address ethical issues arising from increasing volume of international collaborative health research.

### Kenyan health research system

Global health research in Kenya is mainly undertaken through the national health research system, comprising of various players coordinated by the Ministry of Higher Education, Science and Technology (MOHEST) through the National Council for Science and Technology (NCST) (See additional file 1). The main players include the Kenya Medical Research Institute (KEMRI) through its ten centres, private and public hospitals and universities, research networks and nongovernmental organisations both local and international.

### Data Collection and analysis

This work is based on 52 in depth interviews conducted with respondents drawn from institutions involved in global health research in Kenya. These institutions were identified from directorates and discussions with the National Council for Science and Technology, which is the statutory body that authorises and grants clearance to undertake research in Kenya. We therefore aimed to speak to heads of these institutions and in the case of researchers, the heads of departments who could provide an overall voice in terms of the research undertaken. Our recruitment decisions were based on the potential of respondents to give a rich, deep and thorough account of how the benefits arising from global health research was decided. Our main focus therefore was people who could authoritatively speak on behalf of such institutions that are involved in making such decisions. Based on this, we focused on interviewing the leaders of organised groups that are called upon to facilitate the conduct of health related research. Respondents were drawn from six categories (see table 1) representing the actors expected to continually engage with ethical issues arising from the conduct of global health research. In the case of the community representative groups, we relied on the already existing community liaison arrangements under respective research institutions.

**Table 1:** Institutions from which respondents were recruited

Participating institutions	No. of Interviews
Research institutes & University depts. with health science departments/schools/institutes	16
NGOs with research functions	3
Policy makers	4
Ethical review bodies & Research coordinating bodies	11
Pharmaceutical Firms & Other funding bodies	7
Civil Society Organisations (CSOs) & community representative/Advisory groups	11

Potential respondents were contacted by telephone, to discuss the study objectives and inclusion criteria. They were later invited to participate in the study, and arrangements for formal interview made accordingly. Interviews were conducted face-to-face by the principle investigator PI, except for a few that were conducted by telephone, and the final sample was determined by the point of saturation. All interviews except those with members of community representative groups were conducted in English, following an interview guide. Six interview guides were used corresponding to the category of respondents as described in table one. The guides were piloted, revised and those for the community representative groups translated into Kiswahili. These interview guides are available on request.

Interview questions focused on interviewee's familiarity with the concept of benefit sharing and the description of the current practice as well as what ought to happen. The data regarding stakeholder familiarity with the concept of benefit sharing is however the subject of a separate manuscript that is under review. The definition of benefit sharing was not immediately provided to the respondents but discussions over benefits were framed alongside the conceptualisation of benefit sharing provided by Simm Kadri 2005 (ibid) as dealing with concerns over "*what, if anything, should be provided to research participants (both as individuals and groups), and by who, after their participation, and what, if anything, should be made available to others in the host community or country during and after completion of the research"*

Interview discussions were audio recorded, transcribed into MS Word (Microsoft Corporation) and imported into NVivo 8 software [43] that was used for the indexing, structuring and theorising during the analysis. Interview transcripts were read by all investigators to identify meaning units and develop an initial organising system for data coding. This organising system was developed following a criterion suggested by Srivastava and Hopwood, [44], which involved exploring data alongside issues people were talking about, the original issues that the researcher set to find out and the contradictions between the two. Analysis proceeded from open coding, constant comparison and coding into each other to the point of saturation and later organising the codes into themes. We have used quotes from different stakeholders to illustrate theme and those themes that are derived from multiple perceptions.

### Ethical Considerations

The study was approved by the Kenya National Ethics Review Committee (NERC). Informed consent was obtained from all respondents, including permission to record the conversation. The interview data presented here is anonymised by assigning pseudonyms and attribution only made to the broad category from which the respondents were recruited to ensure the confidentiality of respondents.

## Results

For simplicity we present stakeholder's views regarding the benefits of global health research in three categories namely, benefits to those taking part in research including researchers and participating local institutions, benefits to the wider community in which research is located and lastly the societal benefits that are associated with successful completion of research.

### Benefits to those taking part in research

The first category includes benefits mainly accruing to participants, researchers and the host institutions.

#### (i) Access to investigational products and care

*"... I think there are several things that come at an individual level/.../during the time they are in the study you follow them up, you provide them with free medical care for the period they are in the study this one I would say it is a benefit... taking care of their medical care in our setting is something that most of us cannot afford especially in our rural areas" (Researcher 5)*.

Access to interventions that were being developed through research was generally regarded as an ideal form of benefit that should be provided to individuals directly participating in research. While the above response is mainly biased towards clinical research, it was apparent this view was held even where research involved non medical interventions like training.

Besides access to research interventions participants also expect to benefit from access to free or improved medical care more generally, especially through follow-up by clinicians. These were seen by some as key benefits for several reasons. First, the fact that most people participating in research cannot afford routine care, and second that care within a research context is of superior quality compared to the prevailing standard of care as illustrated by the quote from a respondent drawn from a public private partnership.

*"... I think compared to normal treatment standards, patients have a better opportunity...they will see a Doctor more often, they will be followed up closer than if they come with Malaria to their local hospital and get the standard treatment". (Public private partnership2)*.

#### (ii) Compensation for time and effort

*"... But you see, it is almost impossible to call somebody to walk from some place to come to the other place and you don't like give them something. So incentive is really something in African study or culture ... "(Researcher 1)*.

Compensation for time and effort for those participating in research was identified by several respondents, representing a cross section of stakeholders, as a form of benefit that should be made available. A related concern was the demand for reimbursement of direct costs incurred while participating in research. Those supporting compensation as a form of a benefit argued that incentives and rewards were critical for motivating participation

Other forms of benefit sharing among those participating in research were mainly seen as advantageous to local researchers and institutions.

#### (iii) Technology transfer

*"... we cry for transfer of technology and as a way of addressing some of these, and that is what we normally insist on, to try to get some of our people as a Committee, before we approve a protocol, to be trained in these things...we try to look at it and say what is there in it for Kenya in terms of transfer of technology as a benefit for the country..." (*IRB member 4*)*

Some IRB members attached great significance to technology transfer because it was seen to be of great importance to the host country. Different examples of technology transfer including training and mentorship of local researchers by collaborating partners from the north were given as examples. Actual technologies were also mentioned including laboratory equipment and techniques for various types of research.

*"...when you are mentored by somebody who knows how to do those particular things after sometimes you will be able to write your own proposal and also because for example you are writing this with/.../and he is respected, then they/.../might actually also respect it (the name of a local mentee)" (Researcher 4)*.

Opportunities for mentorship were deemed important in areas like publishing, definition of relevant research questions and the preparation of high quality research proposals capable of competing internationally for funding as illustrated by the following quote by a member of an IRB;

*" ... They are starving for funds; they are starving for publications, they starving for name, and so they collaborate, come, and we'll do it" (IRB5)*.

#### (iv) Brain gain and retention of qualified personnel

*"... he likes to talk about 'brain gain' as opposed to 'brain drain'. I mean the fact is, we are being able to provide interesting research work for lots of scientists.... but there are a lot of Doctors that are able to do what they want to do which is to stay in their own country and work in their own country" (Public private partnership1)*.

Several respondents also noted that global health research provides employment opportunities for local scientists. The quote by public private partnership 1 uses the metaphor of brain gain to illustrate how global health research contributes to retention of qualified people who might otherwise be lost to developed countries in the absence of local opportunities. The value of retaining qualified personnel locally was articulated in terms of their contribution to the local health care systems.

#### (v) Infrastructural development

"... Some of the equipments here are extremely old and some of the ones that have been bought recently are mainly because of the projects, otherwise the centre would have collapsed by now" (Researcher 2)

Besides the support given to individual researchers, other forms of capacity building might include development of the research infrastructure through for instance the provision of equipment to institutions hosting research as illustrated in the above quote (and an idea also linked to the idea of technology transfer).

Respondents gave other examples of capacity strengthening especially for institutions like hospitals which host global health research studies including; (i) expansion of bed capacity, (ii) provision of ambulances, (iii) ICT connectivity and (iv) training of local healthcare workers in basic skills to improve the quality of care. Overall such forms of benefits are regarded as instrumental to improvements in the quality of healthcare services offered in those institutions.

The forms of benefits that are articulated in sections i-iv above mainly aim to benefit research participants, researchers and the host institutions. The emphasis on forms of benefits aimed at local researchers and the local research institutions are clearly evident and the reason given for this was that, strengthening the capacity of the two (local researchers and the research institutions) is a necessary condition for benefiting the host countries.

### Benefits to the wider community

The second broad category of forms of benefit sharing within global health mainly targets the wider community in which research is undertaken. Although research participants, researchers and strengthening of local institutions are a part of the community, this category outlines indirect benefits whose enjoyment is not based on direct involvement in research but rather are in keeping with the demands of social justice.

#### (vi) Provide social amenities

*. "...we want to ensure that if you are carrying research within a particular community, you need to give them something back for instance a hospital or something, even if you are doing clinical research. It can be anything else that can benefit the community. Some sort of infrastructure, so that they can at least see that they are benefiting. Once you have done that, actually they will feel that they are part of the process..." (Civil Society Organisation 1)*.

Some respondents also suggested that provision of social amenities be considered a form of benefit to the community. The metaphor of giving back was used variously while appealing to the need to consider the broader interests of the host community while making decisions over which benefits to provide.

The rationale for providing such amenities as a valuable public good is illustrated by the following quote by a respondent from a civil society organisation

"*... these people can at least feel that actually even though we are not part of this research, (meaning not directly participating in the research) our lives have also improved to some extent, even though I did not get money as an individual, I can see a nice school somewhere, I can see a dispensary, I can see a water point, things like that*". *(Civil Society Organisation 3)*

#### (vii) Community mobilisation

"...So the benefits here, let's say, is that the women or the community no longer think of going to the traditional medicine men. They now know that "if my child has epilepsy, I will go to KEMRI..." (*KEMRI Community Representative *3).

Global health research is also expected to mobilise communities towards positive changes in health seeking behaviour. Several community representatives' groups noted during the interviews that communities often acquire better knowledge of disease conditions being researched when they take part in research, and this contributes to positive changes in attitudes and practices towards health promotion.

The following quote by a member of a community representative group further illustrates a case of positive mobilisation;

*"...I think the biggest benefit I have seen is the reduction in malaria cases, because this disease to be honest had killed many people. The cases reduced, and all was because those researchers taught us how to use the nets.../.../some members of the community did not even know what causes malaria..." (KEMRI Community Representative 2)*.

#### (ix) Other non research related assistance

"*... So there is a programme already in place that benefits them, pays for their school fees, takes them to school, ploughs their land and plants, give them fertilizer. So you see this entire OVC (Orphans and Vulnerable Children) package has benefits and all we're asking from that particular population is just to see what the outcomes are*" *(Researcher1)*.

Global health research is also associated with other benefits that are not directly related to research. The above quote for instance illustrates how some researchers respond to pressing needs with benefits to the wider community in return for participation of specific groups in research. Interestingly, these forms of benefit sharing were not directly related to the research but were defined by the community needs.

### Societal benefits

The last category of forms of benefit sharing contains benefits that are aspirational in nature and therefore mainly result once research is complete.

#### (x) Availability of medical and public health tools

"*...One of the issue is we get new products. I think that is a good thing because we get to get new products and we can be able to prevent much more diseases that we have been dealing with...*" *(Policy Maker 3)*.

Global health research was regarded by some as an opportunity for developing future medical and public health tools for resource poor settings. Access to such medical and public health tools was further considered beneficial in terms of the potential for research evidence to inform policy and delivery of health care services, suggesting some value given to research whose final outcome is generation of knowledge broadly defined. Such views acknowledge the potentially trans-generational aspects of research benefits.

#### (xi) Access to proven interventions

"*...I think what we should be asking ourselves is that okay, "We have several candidates being put in place if one of them become successful how do we get access to this vaccine and I think that should be the bigger question that people should be asking...*" *(Researcher 5)*.

Apart from providing the opportunity to develop medical and public health tools, global health research is also expected to promote access to such interventions. The above quote explicates this expectation, suggesting that access to the actual products developed through research is an important benefit.

## Discussion

We have presented data illustrating different forms of benefits that are associated with global health research undertaken in resource poor settings. The forms of benefits are presented along three broad categories that largely resonate with issues of justice that have dominated debates within global research ethics for the last decade. While the forms of benefits that are listed here do not substantially depart from what has been proposed in the theoretical literature [27,28,45,46] ours represent a first attempt to empirically explore the range of benefits that stakeholders in resource poor setting might expect when global health research is undertaken in their setting. More importantly however, the forms of benefits provide a lens through which to reflect on the social values that may be expected to inform the ethical acceptability of global health research in poor settings.

The range of benefits articulated here represents a combination of both micro and macro level concerns for justice by, for instance, seeking to engage with interests of those facilitating research, and the broader systemic issues that make resource poor settings vulnerable to exploitation. The demand for access to products under investigation is an acknowledgement of the need for research to engage with current crises that face people in resource poor settings including lack of access to medical care. In addition, the demand for compensation is seen here as addressing commutative justice which perhaps recognises the contribution made by those facilitating research [47].

In most cases, some forms of benefits that are mentioned here have been contentious. For instance, compensation as a form of benefit has been a subject of longstanding theoretical debate within global research ethics. Demands for compensation have been debated broadly as: i) appreciation, ii) compensation and iii) incentive payments [48]. A key concern is whether compensation induces people into participation [48,49] or whether it even amounts to coercion [50,51]. Some bioethicists have also claimed that compensation payments might compromise scientific integrity of research since participants may withhold information that threatens their participation [50,52], while others worry that it will kill altruism and make research participation a commodity to be traded [53]. The potential of payment to be coercive has however been refuted by others, who see the claim as false and incoherent [54]. Several models have also been developed to address the potential for inducement, including the wage payment model [49,55], the market model [56] and the reimbursement model [57]. What is clear from our work is that participants do have expectations and that further empiric work could inform how to select the most appropriate forms of compensation.

On the other hand, the provision of collateral benefits such as improving health care, provision of social amenities or community mobilisation have also been criticised as a case of holding global health research to a different ethic only applicable to health care provision [58] and one that essentially seeks to correct global inequality [59].

## Conclusion

Contrary to the categorisation of research benefits into those addressing micro or the macro level issues of justice, our empiric findings suggest that both are relevant. Our findings clearly suggest that global health research is expected to engage with the material conditions that define settings in which the research is undertaken. Convincing cases for all the forms of benefits and the diverse nature of intended beneficiaries can be made. Embracing all these forms of benefits however presents a challenge over just how the various competing interests/needs can be balanced ethically, especially in the absence of structures to guide the process. Instead of providing a definitive solution to this challenge our findings should point to the need for greater dialogue to facilitate value clarification among stakeholders involved in global health research. It is clear that work will be required on a broader question to clarify which forms of benefits, under which situation could be justified from a normative perspective.

## Competing interests

The authors declare that they have no competing interests.

## Authors' contributions

The preparation for and conduct of the study was undertaken by all authors. LGM conducted all the interviews, and undertook the initial analysis supported by MP, RF and ME. All authors contributed to the development of the final manuscript and approve the final version.

## About the author

Lairumbi Mbaabu is a research training fellow at the KEMRI/Wellcome Trust Research Programme. His research interests are in global research ethics, community engagement and health systems research.

Michael Parker is Professor of Bioethics and Director of the Ethox Centre at the University of Oxford. His main research interest is in the ethics of collaborative global health research.

Raymond Fitzpatrick is Professor of Public Health and Primary Care at the University of Oxford. He is responsible for teaching medical students, contributing to a master's degree in Global Health Science and supervising students doing doctoral-level health research. He has for many years pursued research focused upon evaluating the quality and impact of health services from the perspectives of patients and users of services.

Michael English is Professor of International Child Health, and Senior Research Fellow at the KEMRI/Wellcome Trust Research Programme.

## Supplementary Material

Additional file 1 **National health research system in Kenya**. The file shows the institutions that undertakes health related research in Kenya, that together constitutes the national health research system. Click here for file
