# Supplementary figures and images for: Forms of benefit sharing in global health research undertaken in resource poor settings: a qualitative study of stakeholders' views in Kenya
Source: Philos Ethics Humanit Med. 2012 Jan 17;7:7. doi: 10.1186/1747-5341-7-7 (PMC3274462; doi:10.1186/1747-5341-7-7)

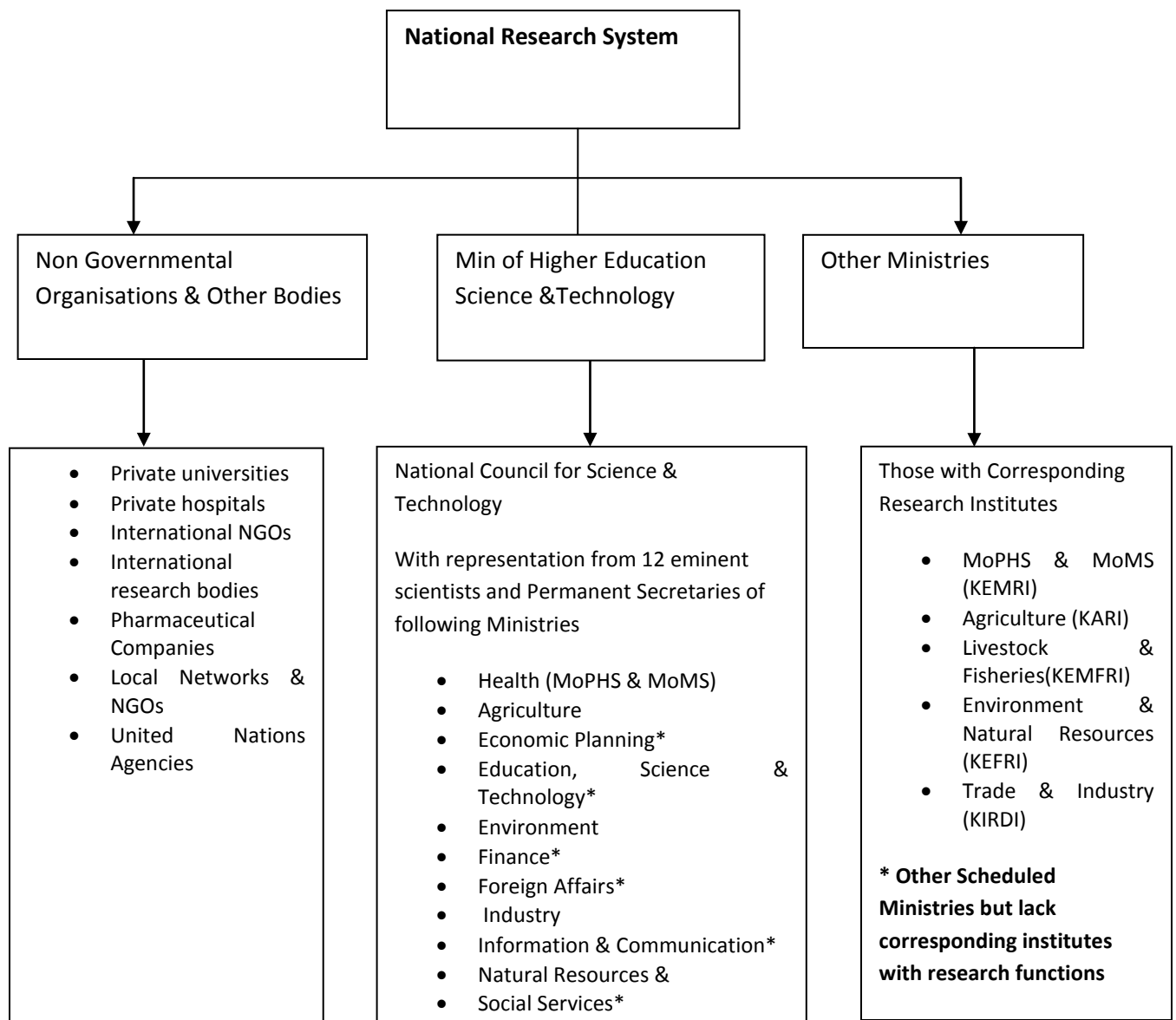

Supplement: Additional file 1 — National health research system in Kenya. The file shows the institutions that undertakes health related research in Kenya, that together constitutes the national health research system. [file 1747-5341-7-7-S1.PDF]
